# Neurotropic Enterovirus Infections in the Central Nervous System

**DOI:** 10.3390/v7112920

**Published:** 2015-11-24

**Authors:** Hsing-I Huang, Shin-Ru Shih

**Affiliations:** 1 Research Center for Emerging Viral Infections, Chang Gung University, Kwei-Shan, Tao-Yuan 33302, Taiwan; hihuang@mail.cgu.edu.tw; 2 Department of Medical Biotechnology and Laboratory Science, College of Medicine, Chang Gung University, Kwei-Shan, Tao-Yuan 33302, Taiwan; 3 Graduate institute of Biomedical Sciences, College of Medicine, Chang Gung University, Kwei-Shan, Tao-Yuan 33302, Taiwan; 4 Clinical Virology Lab, Departments of Laboratory Medicine, Chang Gung Memorial Hospital, Kwei-Shan, Tao-Yuan 33305, Taiwan

**Keywords:** enterovirus, central nervous system, neurotropism, pathogenesis

## Abstract

Enteroviruses are a group of positive-sense single stranded viruses that belong to the *Picornaviridae* family. Most enteroviruses infect humans from the gastrointestinal tract and cause mild symptoms. However, several enteroviruses can invade the central nervous system (CNS) and result in various neurological symptoms that are correlated to mortality associated with enteroviral infections. In recent years, large outbreaks of enteroviruses occurred worldwide. Therefore, these neurotropic enteroviruses have been deemed as re-emerging pathogens. Although these viruses are becoming large threats to public health, our understanding of these viruses, especially for non-polio enteroviruses, is limited. In this article, we review recent advances in the trafficking of these pathogens from the peripheral to the central nervous system, compare their cell tropism, and discuss the effects of viral infections in their host neuronal cells.

## 1. Introduction

Enteroviruses belong to the *Picornaviridae* family. This genus consists of 12 species that include enteroviruses A to J and rhinoviruses A to C. Some enteroviruses have been shown to have the ability to infect the central nervous system and cause various neurological symptoms, such as encephalitis, meningitis, paralysis and ataxia. Among these viruses, poliovirus is the most well-known pathogen because it can cause poliomyelitis in infants and young children. In addition, other non-polio enteroviruses accounted for approximately one half of aseptic meningitis cases in children [1]. However, the precise mechanisms associated with these manifestations remain largely unknown. Although many enteroviruses can invade the nervous system, most of their trafficking routes are unidentified. Furthermore, knowledge about their CNS host cells and the consequences of disease progression is limited. Therefore, it is difficult to develop an effective therapeutic strategy against the neurological infection that was caused by these neurotropic enteroviruses. In this review, we focus on these viruses and discuss their trafficking mechanisms, the factors related to neurotropism and neurovirulence, and the neuropathogenesis associated with neurotropic enteroviral infections.

## 2. Neurological Diseases Associated with Neurotropic Enteroviruses

Most enterovirus members only cause mild symptoms, such as hand-foot-and-mouth disease, herpangina, pleurodynia, rashes, and rhinitis. However, some enteroviruses have been discovered to have the ability to infect the central nervous system and result in various neurological diseases including aseptic meningitis, encephalitis, paralysis, and ataxia [2]. Meningitis is an acute inflammation of the membranes covering the brain and spinal cord. Meningitis that is caused by virus infections occurs often in children. Coxsackie and echovirus are the most common causes for viral meningitis [3]. However, other viruses such as poliovirus and enterovirus 71 (EV71) have also been demonstrated to have the ability to evoke aseptic meningitis [3]. Coxsackievirus, poliovirus, and EV71 have been demonstrated to induce encephalitis in infected patients [4,5,6]. Although encephalitis has not frequently appeared in enteroviral infections, EV71 is one of the most common enteroviruses that have been isolated from the samples collected from children with viral encephalitis [5]. Coxsackievirus infection is associated with meningoencephalitis, a neurological disease characterized by inflammation of both the meninges and brain [7]. In addition to coxsackievirus, EV71 and echovirus 9 have also been reported to be associated with acute meningoencephalitis [8,9].

It is well known that a poliovirus infection can result in poliomyelitis. However, research has demonstrated that in addition to polioviruses, other enteroviruses, such as EV71 and enterovirus D68 (EV-D68), can result in polio-like paralysis [10,11,12,13]. Although the poliovirus vaccine has successfully eradicated most poliovirus-infection-associated diseases globally, the increased incidence of flaccid paralysis caused by a nonpolio-enterovirus infection is highly noticeable. Acute flaccid paralysis (AFP) was frequently observed among EV71 patients [14]. The association of EV71 infection and AFP has been demonstrated in several studies [15,16,17]. Thus, EV71 has been considered as an important pathogen for AFP. In 2014, mysterious outbreaks of AFP were reported in North America. EV-D68 was detected in the specimens that collected from the patients with AFP [18,19]. It is possible that EV-D68 infection may contribute to the onset of AFP. However, the link between EV-D68 infections and AFP is not fully established. A recent study suggested there is another new virus named enterovirus C105 could be the causative pathogen of AFP outbreak in 2014 fall [20]. Most patients with flaccid paralysis only partially recover, and some of them show persistent weakness. Furthermore, other enteroviruses, such as coxsackievirus and echovirus, were also detected in flaccid paralysis patients, although their incidence is low [21]. Guillain-Barre syndrome (GBS) is an inflammatory pathologic condition; the typical sign for this disease is acute inflammatory demyelinating polyradiculoneuropathy, which is usually present days after respiration of the gastrointestinal illness. It has been suggested that GBS may serve as a cause for AFP and, thus, it may contribute to EV71 infection-associated AFP [22].

Previous studies showed some patients who recovered from acute poliovirus attack may develop post-polio syndrome (PPS), a neuropathological condition associated with the symptoms including muscle weakness and muscular atrophy [23,24]. In contrast to poliovirus, the neurological complications that resulted from a non-polio enteroviral infection have been deemed to be acute and transient. Except in cases of death, most patients recovered without neurological sequelae. However, recent studies have noted that long-term neurological manifestations could be observed in patients who experienced non-poliovirus infection, resulting in serious neurological symptoms. For example, Chou *et al.* [25] demonstrated that an enteroviral infection may be associated with the increased risk of attention deficit hyperactivity disorder (ADHD). Among those patients who survived from EV71 infection-associated serious neurological diseases, neurological disorders such as ADHD, delayed neurodevelopment, and reduced cognitive functioning were observed [26,27]. Furthermore, infection by coxsackievirus during childhood seems to be able to increase the risk of schizophrenia [28]. Nevertheless, the causes of these neurodevelopmental and behavior disorders are not totally clear and currently there are no sufficient evidences to prove that the enteroviruses are responsible for these diseases.

## 3. Invasion of the Central Nervous System

A common property of neurotropic enteroviruses is their ability to invade the CNS from primary infection sites. These viruses are replicated in the gastrointestinal tract first and then disseminated to other organs/tissues via specific pathways. To understand how these viruses gain access into the nervous system is important for us to understand their pathogenesis and to develop strategies to reduce the incidence of neurological complications. Intensive studies were performed to elucidate the routes of poliovirus that infect the CNS. The results showed that a poliovirus infection can spread into the CNS through neuromuscular junctions [29]. The retrograde transport model, which is a pathway that transports viral particles from the muscle to the CNS, has been proposed because muscle injury can enhance poliovirus neuroinfection [30,31]. In addition to the poliovirus receptor-dependent retrograde axonal transport pathway, it has been proposed that poliovirus can efficiently reach the CNS in a receptor-independent axonal transport process [31]. However, other studies revealed that poliovirus can infect cultured human brain microvascular endothelial cells (HBMEC), which indicated that this virus may directly cross the blood-brain barrier. Furthermore, virus particle binding with receptors on the endothelial cells is able to induce activation of a protein phosphatase to facilitate virus entry [32]. Therefore, multiple routes may be used for poliovirus to enter the nervous system. In addition to poliovirus, other enteroviruses, such as EV71 and coxsackievirus, can also utilize the retrograde transport system to invade the CNS. Chen *et al.* [33] demonstrated that EV71 transmitted into the nervous system occurs primarily via the retrograde axonal transport route in mice.

Many neurotrophic viruses infect leukocytes and use them as vehicles for transportation into the CNS, which is known as “Trojan Horse” entry. For example, respiratory syncytial virus proteins can be detected in the peripheral blood mononuclear cells (PBMCs) isolated from infected mice on days three and seven post-infection; blockage of the transendothelial migration of leukocytes can reduce the viral load in brain tissues [34]. Previous studies have shown that monocytes can support poliovirus replication and that the neurovirulent poliovirus replicates faster in monocytes [35,36]. Therefore, it has been suggested that monocytes may serve as the poliovirus carrier from the blood stream toward the nervous system [37]. However, there is no direct evidence indicating that poliovirus-infected monocytes are responsible for its CNS invasion. In addition, coxsackievirus can efficiently infect blood-derived Mac-3^+^ mononuclear cells, cells with myeloid morphology and possess the ability to migrate toward the CNS, which subsequently enter the brain through the choroid plexus epithelium [38]. Since EV71 has been demonstrated to be able to infect various immune cells such as CD14^+^ cells, dendritic cells, and PBMCs, it is possible that EV71 may use these immune cells as vehicles to spread to other tissues/organs [39,40,41]. Other non-polio enteroviruses, such as echovirus, have been reported to be able to infect dendritic cells [42]. However, to date, there is still no evidence to support that these viruses utilize the infected immune cells to invade the CNS.

## 4. Tropism of Neurotropic Enteroviruses in the Nervous System

Different enteroviruses target different areas of the nervous system. Poliovirus can be detected in the anterior horn of the spinal cord, which contains motor neurons that affect skeletal muscles [43]. The replication of poliovirus in motor neurons within the spinal cord causes poliomyelitis [43,44]. However, EV71 targets other areas of the central nervous system. Lesions were found in the spinal cord, cerebellum, medulla, pons, brain stem, and cortex according to clinical observations [45]. The neurons located in the medulla oblongata, brain stem, and cervical spinal cord have been implicated with the onset of neurogenic pulmonary edema [46]. However, whether the neurological pulmonary edema is a consequence result from EV71 infection in brain stem and medulla oblongata has not been elucidated. Intracerebral infection of neonatal mice with echovirus type 1 (EV1) results in paralysis of the hindlimbs. EV1 viral RNA could be detected in retrosplenial and motor cortices, where the vacuolization and necrotic cells were observed, within the brains of paralyzed mice [47]. The lesions in the motor cortices may be responsible for the spastic paralysis of the EV1 infected animals. In an established coxsackievirus B3 CVB3 animal model, the virus can be widely detected in the brain. The lesions could be observed in the cortex and the hippocampus regions [48]. Another model used one-day-old mice for an intracranial infection, and the results showed that the virus was expressed in the choroid plexus and the subventricular area, where are rich with neural progenitors [49]. The CVB infection of neural progenitor cells may explain the neurodevelopmental deficits that due to infection of fetus [50]. Intriguingly, although EV-D68 is an emerging enterovirus that may cause polio-like syndromes in young children, magnetic resonance imaging (MRI) results showed that there is no enhancing lesion in either the gray matter of the spinal cord or the brainstem upon EV-D68 infection [51].

## 5. Susceptible Cells for Neurotropic Enteroviruses

Identification of the natural targeted neuronal cells of enteroviruses is limited by the accessibility of human specimens; thus, most studies are performed using neuroblastoma and glioblastoma cells. Currently, our knowledge about the host neuronal cells of neurotropic enteroviruses is based on the data collected from animal experiments and autopsy cases that provide clues to elucidate their possible CNS target cells *in vivo*. In a transgenic mouse model, the presence of a poliovirus antigen could be detected in motor neurons in the anterior horn of the spinal cord, pons and medulla oblongata [52]. The data collected from coxsackievirus B1, B3 and B5 infected suckling mice revealed morphological changes in the medulla oblongata and necrosis in the neurons [53]. Several studies have applied primary murine neuronal cells or differentiated human neuroblastoma cells as host cells to confirm the susceptibility of candidate neuronal target cells *in vitro* to study the effects of viral infections. Yoon *et al.* [54] demonstrated that CVB4 can infect primary rat neurons. Furthermore, Coxsackievirus B has also been shown to be able to infect mouse primary cortical neurons [55]. In addition to neurons, astrocytes are permissive to CVB infection [56]. The immunohistochemical staining results of brain tissue collected from patients who died from EV71 infections showed that viral antigens could be detected in neurons [57]. In murine-adapted EV71 infected mice, the EV71 antigen and RNA could be detected in the neurons, thus confirming that neuronal cells are susceptible to EV71 infection [58].

Recent studies have shown that CVB3 not only infected neurons but also existed in the neural progenitors residing in the brain [50]. Neural progenitor cells are primitive cells with abilities in differentiating all types of neural cells. *In vivo* experimental results revealed that proliferating neural progenitors were targeted by CVB3. Further studies showed that CVB3 is able to complete the replication cycle in the host neural progenitors [59]. In addition to CVB3, EV71 is capable of infecting neural progenitor cells [60]. However, although CVB3 can infect differentiated neural progenitor cells, diminished expression of viral protein and decreased viral replication were observed [59]. Similar results were observed in EV71 because differentiated neuronal and glial cells are less permissive than their undifferentiated progenitor cells [60]. The varied viral replication rate could be attributed to the different expression levels of virus receptors and/or the presence of cell-specific factors that can modulate viral growth. The receptors utilized by EV71 and CVB3 to enter the neural progenitors have not been elucidated; further studies should, thus, be performed. It has been proposed that the cell cycle status may affect virus replication [61]. CVB3 is preferentially targeted to proliferating neural progenitor cells; thus, it is possible that the active replication of progenitor cells makes them easy targets for these viruses.

## 6. Receptors Associated with Neurotropic Enterovirus Entry

Receptors mediate the entry of viruses into the host cells and, thus, play essential roles in cell tropism. Through intensive studies, many enterovirus receptors have been identified. Cluster of differentiation 155 (CD155) serves as a receptor in mediating poliovirus entry on target cells [62]. CD155 mRNA can be detected in various tissues including small intestine, brain, skeletal muscle, lung, liver, and kidney [63]. A recent study demonstrated that CD155 is expressed within embryonic tissues that subsequently raise spinal cord motor neurons, which may explain the cell tropism of poliovirus [64]. Further study showed that CD155 is expressed in the motor neurons and at the end plates of muscle cells, thus implicating its role in mediating virus entry to motor neurons [65].

Echoviruses and Coxsackie B viruses use human decay-accelerating factor (DAF, CD55) as their receptors to enter host cells [66,67]. DAF expressed at the apical surface of polarized Caco-2 cells is attributed to virus transport [68]. DAF is also expressed by neurons and, thus, they may mediate the entry of echoviruses and coxsackieviruses into neuronal cells [69]. However, recent study performed showed not all CVB isolates can bind to DAF and, thus, DAF is not the required receptor for all coxsackie B viruses [70]. In addition to DAF, coxsackievirus uses other receptor such as coxsackievirus and adenovirus receptor (CAR) to infect the target cells [71]. Interestingly, CAR may directly link to the endocytic pathway in motor neurons [72]. Salinas *et al.* [73] demonstrated that CAR ligands can trigger CAR-CAR disengagement, promoting internalization. Therefore, a virus may mimic the ligand of CAR and promote the endocytosis of viral particles. For example, CAV-2 and HAdV5 have shown the ability to trigger CAR internalization in various neuronal types [65]. However, whether CAR renders the entry of coxsackievirus in neuronal cells is not clear. A previous study showed that mature neurons were less susceptible than immature neurons to CVB5 infection, and this phenomenon occurred due to the decreased expression of CAR, which is the major receptor for CVB5, in mature neurons [74]. Several receptors that are responsible for EV71 entry have been characterized. These receptors include scavenger receptor B2 (SCARB2), P-selectin glycoprotein ligand-1 (PSGL-1), annexin A2 (Anx2), sialylated glycan, dendritic cell- specific intercellular adhesion molecule-3 grabbing nonintegrin (DC-SIGN), and heparin sulfate [75,76,77,78,79]. SCARB2 is a type III transmembrane protein that participates in the membrane transport process [80]. SCARB2 is widely expressed in most tissues and is deemed to play major roles in establishing EV71 infection. A human SCARB2 transgenic mouse can be infected by EV71 clinical isolates and develop similar symptoms [81]. SCARB2 is highly expressed in neurons of the central nervous system, and a recent study showed that an EV71 antigen could be detected in SCARB2 -positive neuron in an autopsy case of EV71 infection with acute neurological diseases. Not all EV71 strains can bind PSGL-1; the protein exists as a dimer on the surface of various types of immune cells. The expression of PSGL-1 was also observed in neurons and glial cells [82]. Annexin A2 is a pleiotropic protein that is expressed in various tissues including the brain. A recent study showed that annexin A2 is highly expressed in neurons located in the brain [83]. However, the receptors that play a major role in determining the neurotropism of an EV71 infection are not clear. In summary, the viral receptors not only determine cell tropism but may also contribute to the transport of virions.

## 7. Cytokine and Chemokine Production in the CNS upon Enteroviral Infection

Although the CNS has been considered to be an immunologically privileged area, immune responses could be evoked in response to an enterovirus infection. Immune cell infiltration is frequently observed in patients with neurological complications. Histochemical staining reveals that perivascular cuffing and neutrophil infiltration is often present. The histopathology of poliovirus patients showed neuron dropout in the anterior and posterior horns [84]. Furthermore, lymphocyte infiltration was noted in the CNS for two to three weeks following paralytic symptoms that appeared in an infected mouse [85]. However, knowledge about the immunological responses in response to a poliovirus infection in humans remains unclear. Furthermore, EV71 infection results in the infiltration of large amounts of neutrophils in the grey matter with lymphocyte cuffing in patients who died from neurological complications [86]. For CVB3, recruitment of immune cells in the brain was evidenced by animal experiments [38]. The infiltration of leukocytes has been shown to contribute to neuroinflammation [87,88]. Thus, to block the action of CD146, an adhesion molecule expressed on endothelial cells, is able to ameliorate the neural inflammation by limiting the trafficking of leukocytes [89].

The migration of immune cells may be explained by the chemokine production in the CNS during an enteroviral infection. High levels of type I interferons, monocyte chemoattractant protein-1 (MCP-1), interferon γ-induced protein 10 (IP-10), and regulated on activation, normal T-cell expressed and secreted (RANTES), were produced in the CNS upon CVB3 infection [90]. The up-regulated expression of chemokine (C-C motif) ligand 12 (CCL12) may be related to the recruitment of nestin-positive myeloid cells across the BBB and into the CNS [38]. Other chemokines such as chemokine (C-C motif) ligand 4 (CCL4), chemokine (C-C motif) ligand 7 (CCL7), chemokine (C-C motif) ligand 2 (CCL2), chemokine (C-X-C motif) ligand 4 (CXCL4), chemokine (C-X-C motif) ligand 10 (CXCL10), and chemokine (C-C motif) ligand 9 (CCL9) were also increased in the brains of CVB3-infected mice [49]. In addition to chemokines, a recent study showed that a deficiency of interleukin-10 (IL-10) can reduce lymphocyte infiltration to the brain during cytomegalovirus infection via up-regulating the expression of CXCL9 and CXCL10 [91]. Upon EV71 infection, IL-10 production was increased in patients with pulmonary edema [92]. However, whether IL-10 contributes to EV71-induced neuroinflammation still needs to be determined.

Various cytokines were produced during an enterovirus infection. In addition to playing roles in triggering immune responses to control the infection, the secreted cytokines may exert deleterious effects on neighboring cells. Upon stimulation with a poliovirus antigen, the production of IFN-γ was produced and could be measured in whole blood [93]. The neurovirulence was increased in poliovirus infected type I IFN receptor-deficient mice, which indicated the importance of IFN in poliovirus-induced neuropathology [94]. However, knowledge about other cytokines involved in poliovirus infection-induced acute neurological diseases remains limited. Previous studies have shown that the increased production of proinflammatory cytokines including interleukin-6 (IL-6), IL-1β, and TNF-α are associated with the severity of EV71-induced neurological diseases [95,96]. Furthermore, previous studies showed that increased levels of IL-6, IFN γ, and IL-1 β in cerebrospinal fluid (CSF) are observed in patients with pulmonary edema [95,97]. An animal study demonstrated that the enhanced IL-6 production is correlated with the disease severity and administration of anti-IL-6 neutralizing antibodies can improve the survival rates [98]. Furthermore, treatment EV71 infected mice with IL-6 provoked mild pulmonary edema which accompanied by pulmonary dysfunction [99]. However, the mechanisms about the effects of IL-6 in EV71-related neuropathogenesis are not clear. Enhanced expression of IFN-γ has been observed in patients with serious neural complications such as pulmonary edema [97]. Since IFN-γ is capable of increasing vascular permeability, it is likely to contribute to EV71-caused pathogenesis [100]. The cells that are responsible for IFN γ production include helper T cells, cytotoxic T cells, and NK cells. Thus, the infiltrated immune cells in the CNS may serve as sources for IFN-γ secretion. The concentration of CSF IL-1β is significantly higher in patients who suffered from EV71-induced pulmonary edema [97]. Furthermore, IL-1β transcripts could be detected in rat brains. This finding provides evidence that IL-1β can be up-regulated in the CNS. However, the cells responsible for IL-1β production have not yet been identified.

A previous study showed that a variety of cytokines could be detected in the myocardium of CVB3-infected mice even a long time after the primary infection [101]. The existence of cytokines/chemokines is associated with inflammation and may contribute to long-term sequelae. Furthermore, because ultraviolet light (UV) inactivated CVB3 cannot trigger cytokine production, the expression of these cytokines may represent active virus replication in these tissues [102]. Through an analysis that was performed in the CNS of patients with post-polio syndrome, the focal perivascular intraparenchymal chronic inflammatory infiltrates contained most B cells, and rare macrophages were observed. This observation indicates that poliovirus may persist in the brain for a long time and may trigger the secretion of cytokines/chemokines, which attract immune cells to the brain and, thus, are attributed to the pathogenesis [103].

## 8. Persistent Infection of Neurotropic Enteroviruses

It is known that several enteroviruses can establish persistent infection in specific cell types. In patients with post-polio syndrome, poliovirus RNA can be detected in cerebral spinal fluid years after the primary infection [104]. In addition to poliovirus, other enteroviruses also exhibit persistent infection. For example, EV71 could be consistently detected in secretions and excretions even a long period after the initial infection [105]. However, the site for establishing EV71 persistent infection has not been identified. Continued replication of coxsackievirus has been demonstrated in the heart tissue and is related to disease progression. Animal experimental results showed that CVB3 persists in the myocardium [106]. In addition to heart tissue, Feuer *et al.* [90] showed that CVB3 may establish persistence in the CNS.

To establish a persistent infection, multiple viral and host factors may participate in this process. Over a long course of evolution, host cells use immune system to control the infection and viruses develop various strategies to evade cellular defense mechanisms. Some viruses have different strains that carry different nucleotide compositions; thus, some can persist in cells while others cannot. For example, the Theiler’s original group in cardiovirus Theiler’s Murine Encephalomyelitis Virus (TMEV) infects the CNS persistently and results in demyelination, but another group (GDVII) causes acute neurological diseases [107]. Previous studies showed the persist enteroviral infections is related to the selection of defective viruses from viral quasispecies generated under immune selection. Passage of CVB3 in primary heart muscle cells has been shown to create the deletion in the 5′ untranslated region (5′ UTR), and this change was related to the decreased ability in causing cytopathic effects of target cells without changing the efficiency of virus replication [108,109]. The positive strand RNA is synthesized much more than the negative strand RNA during an enterovirus infection [110]. However, the ratio of positive and negative strands decreases when CVB3 is establishing a persistent infection in the CNS [90]. Similar results have been demonstrated in other tissues that are persistently infected by CVB3, which may indicate that the double stranded RNA complex may exist and may assist in establishing persistence [111]. However, whether other enteroviruses use the same mechanism to persistently infect tissue cells is not clear.

Type I IFNs, especially IFN β, plays an important role in limiting the replication of CVB3 [112]. However, high titers of IFN were observed in persistent viral infections and the IFN β seems be major contributor for promoting persistent infection of lymphocytic choriomeningitis virus (LCMV) [113]. Persistent infection can be developed in enterovirus-infected neuroblastoma cells [114,115], and hence can provide valuable *in vitro* models to study the mechanisms of persistent infection. For example, CCF-STTG1 cells, human astrocytoma cells, are persistently infected by CVB3, and the results showing that receptor usage and decreased IFN β production may be attributed to CVB3 persistence [115].

Although the persistence of poliovirus (PV) has been deemed to play an essential role in the pathogenesis of post-polio syndrome, whether the persistent infection of other enteroviruses is associated with neurological sequelae is not clear. Several studies showed that enterovirus sequences could be detected in neuronal cell bodies of the spinal cord of amyotrophic lateral sclerosis (ALS) patients, thus suggesting an association between an enterovirus infection and ALS [116,117]. However, conflicting results reported that an enterovirus infection is not related to motor neuron disease because the 5′ UTR sequence of echovirus, coxsackievirus, and poliovirus cannot be detected in the CNS tissue of patients with amyotrophic lateral sclerosis-motor neuron disease (ALS-MND) [118]. Thus, further studies need to be performed to confirm the association of EV persistent infection and these diseases.

## 9. Enteroviral Infections Induced Apoptosis and Autophagy

The outcome of enterovirus-infected cells is determined by multiple factors such as the nucleotide composition of the virus, the efficiency of viral replication, and cellular factors. The CNS is primarily composed of neural lineage cells including neurons, astrocytes, and oligodendrocytes and non-neural cells, such as endothelial cells and microglia cells. It is well known that neurotropic enteroviruses are able to invade the CNS and infect neural cells. However, the impacts of viral infection on cell fate are not yet clear.

Since the primary human neuronal and glial cells are difficult to obtain, most studies have been performed using neuroblastoma and glioblastoma cell lines. For viruses that can infect murine cells, mouse primary neural lineage cells have been used as an *in vitro* model to examine the effects resulting from neurotropic enteroviral infections. Previous studies have shown that neuronal cells are permissive to poliovirus, EV71, and CVB3 [119,120,121,122]. Various enteroviruses including poliovirus, CVB3, CVB5, and EV71 are known to be able to induce an apoptosis response in host cells [123,124,125,126]. It has been proposed that an enterovirus infection caused apoptosis process functions in a cell-dependent manner [127]. Poliovirus infection triggers apoptosis in HeLa cells only when viral growth is hampered [123,128]. However, in L20B cells, which are the murine L cells expressed in the human poliovirus receptor, poliovirus is able to be efficiently produced when apoptosis occurs [129]. The study of poliovirus-triggered apoptosis processes in different cell lines showed that the induced programming death pathways varied, although comparable viral reproduction was observed in the tested cell lines. The results indicate that poliovirus caused apoptosis programs to work in a host-dependent manner. Poliovirus can infect motor neurons of the brain and spinal cord and subsequently cause death in the infected neurons, which may be implicated in paralytic poliomyelitis [121]. Through the study of poliovirus infection in IMR5 neuroblastoma cells, c-Jun NH_2_-terminal kinase (JNK) is activated soon after poliovirus infection and then triggers the activation of Bax, the proapoptotic protein that belongs to the Bcl family, which causes cytochrome c release from the mitochondria and thus results in cell death [130]. Furthermore, a poliovirus infection can increase cytosolic calcium concentrations in IMR5 cells, which may be related to PV-induced apoptosis [131].

Upon CVB infection, significant apoptosis and productive virus replication were observed in mouse cortical neurons [132]. However, the inhibition of apoptosis seems to have no effect on virus production [55]. This observation is in contrast to the results that were obtained by studying the effects of apoptosis in CVB3 infected HeLa cells, which showed that the production of viral particles was hampered when apoptosis is blocked [133]. CVB3 infection also triggers apoptosis in murine neural progenitor cells, which contribute to the loss of these cells and may be attributed to impaired neurogenesis [134]. A previous study showed that the distribution of Bag-1 was altered in a CVB3 infected mouse heart, and this event may be associated with cell apoptosis [135]. By utilizing HeLa cells as an *in vitro* model to study the associated mechanism, the inhibition of GS3K-β activation is able to block CVB3-induced apoptosis [133]. Whether Bag-1 and/or GS3k-β play essential roles in CVB3-triggered neuronal apoptosis is not clear.

Recent studies have suggested that autophagy can support the replication of CVB3 in host cells, including HeLa and HEK293A cells, human brain microvascular endothelial cells, and cardiomyocytes [136,137,138]. Furthermore, CVB4 infection has been shown to be able to induce autophagy in neurons and the addition of autophagy inhibitors is able to suppress viral replication [53]. However, in CVB3-infected neural stem cells, autophagic signaling is unaffected. In contrast, following CVB3 infection, a decrease in autophagic signaling was observed in neural stem cell (NSC)-differentiated neuronal cells [122]. These results revealed that the ability of CVB in triggering autophagy and the impacts of autophagy to CVB reproduction is dependent not only on cell types but also on virus strains.

Previous clinical observation of EV71infected patients reveals that neuronal cells are susceptible to EV71 infection [57]. Cao *et al.* [120] demonstrated that EV71 is able to complete its life cycle in rat neural cells and induce the apoptosis of infected cells by decreasing the expression of Bcl-2 and up-regulating the expression of Bax and active caspase-3. Based on a comparison of the induced apoptotic process in non-neuronal and neuronal cell lines, Abl kinase is activated only in neuronal cells and may play an essential role in mediating EV71 induced neuronal apoptosis [139]. Another study showed that EV71 infection results in cell cycle arrest through miRNA let-7b expression in human SH-SY5Y neuroblastoma cells. The production of miRNA let-7b may also contribute to virus-induced apoptosis [140]. In an EV71 mouse model, autophagy could be detected in the brain tissues of infected mice and was implicated in facilitating viral replication [141]. Furthermore, a recent study showed that autophagy flux is induced in EV71 infected SH-SY5Y cells, and the results of animal studies revealed that the administration of autophagy inhibitor 3-methyladenine can decrease the viral load in the brain [142]. Moreover, proteomic analysis revealed that several genes associated with the cell cycle and cytokine/chemokine production were differentially regulated in EV71-infected SHSY5Y cells.

## 10. Conclusions

This review focused on the recent advances of enteroviruses with regard to CNS infections and the factors that contribute to their pathogenesis. Different neurotropic viruses use different strategies to migrate from the enteric tract to the CNS. All of the tested viruses are able to infect the neuronal cells, and cell tropism is determined by the expressed receptors that mediate the viral entry. Intriguingly, in addition to the receptors, there are unidentified neural cell specific factors that may affect viral growth. Although several studies have demonstrated that PV and CVB are able to persist in neuronal cells, the associated mechanisms are not clear. Enteroviral infection of neuronal cells may trigger apoptosis and autophagic influx, which are implicated in enterovirus-associated neuropathogenesis. Furthermore, cytokines and chemokines are produced in neural lineage cells upon enteroviral infection, and the release of these immune mediators may play roles in neural disease progression. We noted that many neurotropic enteroviruses such as CVB and EV71 are capable of infecting neural progenitor cells and actively replicating in these cells. Nevertheless, viral infection causes apoptosis of infected neural progenitors and then results in the loss of these cells, which may contribute to the enteroviral infection-associated long-term sequelae such as learning deficits and impaired recognition. However, the factors attributed to the incidences of neurological diseases, the severity and the progression of diseases are not yet clear. Further studies should be performed to address these questions; the obtained results will broaden our understanding of these emerging pathogens and provide great help in developing effective strategies against these viruses.
